# Validation of dispersion model designated for the coke production industry

**DOI:** 10.1007/s10661-021-09007-z

**Published:** 2021-03-30

**Authors:** Jacek Żeliński, Dorota Kaleta, Jolanta Telenga-Kopyczyńska

**Affiliations:** 1 Institute for Chemical Processing of Coal, Zamkowa 1, 41-803 Zabrze, Poland; 2 Dept. of Air Protection, Silesian University of Technology, Konarskiego 22B, 44-100 Gliwice, Poland

**Keywords:** Atmospheric dispersion, Dispersion modeling, Model validation, SF_6_ experiment, Model quality indicators

## Abstract

In the practical application of air protection, diverse dispersion models are used to calculate the concentration of contaminants in the air. They usually involve a universal character, which typically makes them sufficient for use in almost all conditions, with the exception of those clearly deviating from the average. This is especially relevant to industrial objects of large areas, introducing a great amount of heat and mechanical energy into the air. For such cases, the standard models can be extended in order to adapt them to the unusual local diffusion conditions. Next, to be applied in practice, they must have undergone validation to document the correctness of its operation. The article describes the process of validation of the air quality assessment model containing extended procedures to incorporate special factors affecting atmospheric dispersion in a coke industry. The set of statistical indicators, obtained on the basis of SF_6_ field experiment, evaluate its performance. The short comparison with some popular models of general-purpose character and an assessment of the suitability of individual indicators for validation purposes are also presented.

## Introduction

The phenomenon of changes in the dynamics of the dispersion of pollutants in the lowest layers of the atmosphere under the influence of heat released from sources of considerable size is generally known. It concerns urban areas and big industrial sources that emit technological heat to the atmosphere. While the first of these cases has received widespread scientific attention, the latter seems to have gone unnoticed. For this reason, information on the mentioned phenomenon can be found mainly in works devoted to urban areas.

One assumes that in urbanized environments, the dispersion of pollutants takes place within a shallow roughness sublayer ranging from the earth’s surface to a height roughly exceeding twice the average vertical dimension of local terrain obstacles (Kastner-Klein & Rotach, 2004; National Research Council, 2012). Inside this layer, the flow of air and its turbulence are strongly influenced by objects and activities of anthropogenic origin. This is related not only to the existence of buildings and other objects but also to moving vehicles (Christen et al., 2009) and strong sources of heat (Hanna et al., 2011). This sublayer is perceived commonly as the main area of interest when dispersion modeling in an urban environment takes place.

Heat release is of increasing importance in middle-scale calculations for regions and big cities. Indeed, according to the results of the “heat island” phenomenon investigations, anthropogenic heat influences or even dominates the creation of thermal turbulence and appears as the main factor in shaping convective conditions over the heat emission area. The heat island effect is visible when the air temperature over an urbanized territory becomes higher than the temperature over the outskirts. This difference can even reach 12 K, but the difference of a few kelvins is sufficient for inducing this phenomenon (US EPA, 2004).

Research on this subject has been conducted for many years (Garstang et al., 1975; Landsberg, 1981; Oke, 1981) leading to finding the basic features of such area. They can be briefly enumerated as follows:

- the retention of an atmospheric layer with slightly stable or even neutral stability up to 100–300 m above the ground in night conditions (Tapper, 1990),
- an elevated air temperature (Oke, 1995),
- the appearance of a plume of air that is warmer than in a neighboring area on the leeward side (Landsberg, 1981; Oke, 1995),
- the transformation of the air temperature’s vertical profile into a neutral shape (Glazier et al., 1976; Godowitch et al., 1985) accompanied with the formation of elevated inversion (Uno et al., 1992),
- the appearance (under the condition of weak winds) of air mass circulation directed towards the town center in the air layer close to the ground, and in the direction to the outskirts at a higher altitude (Ado, 1992).

For industrial sources, where heat release takes a slightly more complicated character, some aspects of their influence on contaminant dispersion can be highlighted:

- increased diffusivity coefficients, due to thermal effects, increase the dispersion dynamics over the whole area where heat is released. Increased air flow turbulence in such areas has been reflected both in computational simulations (Mirzaei & Carmeliet, 2013; Xie et al., 2013) and in measurements (Huq & Franzese, 2012),
- the plume rise height is increased due to higher buoyancy. This problem has been researched by many authors over many years, with the use of various methods: full-scale data analysis, wind tunnel experiments, and small-scale or numerical simulations (Briggs, 1984; Contini et al., 2011; Freitas et al., 2006; Kozarev et al., 2014; Żeliński et al., 2017). The observations made in these studies reveal a fundamental dependence between plume convection intensity and the temperature of outlet gases,
- this increased plume rise is often multiplied due to the merging of plumes from multiple sources, forcing an overall buoyancy enhancement which is characteristic of the several point emission points arranged in a line, especially when the wind direction is aligned with their long axis of symmetry. These effects are visible both for a set of point sources and for line and area sources (Schulman & Scire, 1980),
- the buoyancy caused by heat introduced into the air has a tendency to aerodynamically counteract downwashes induced by buildings aerodynamic effects, typically resulting in the plume meandering downwards towards the earth’s surface (Schulman & Scire, 1980). The influence of this effect covers a distance of up to 1 km from the source (Paine et al., 2016). Even though the effect of downwash itself is incorporated into the structure of some new-generation models, such as AERMOD, ADMS, and OML, along with the inclusion of calm winds options (Berkowicz et al., 1986), none of them can be adjusted to incorporate the modified aerodynamics of the plume due to increased buoyancy.
- the high moisture content in outlet gases causes, when a plume rises, that the latent heat of condensation is introduced into the air (Hanna & Britter, 2002﻿). This concludes with an increase in plume temperature (although a part of this heat causes secondary, partial evaporation) and an additional plume rise. This creates an effect that is not considered by most dispersion models because of the common assumption concerning a dry character of the plume (Paine et al., 2016).

Most of these factors can be taken into account in dispersion modeling by modifying source characterization data in a manner, which force propagation models to behave as though they have directly taken the heat output into consideration (Paine et al., 2016). For instance, the enhanced population value can be used in order to introduce the urban-rural temperature difference (Oke, 1995). For second-generation models which do take advantage of sensible heat emission information for boundary layer parameterization, the additional heat output can be reflected indirectly by taking into account locally modified land use, or directly by introducing the heat emission value into calculations (Cimorelli et al., 2005). Also mechanical turbulence creates a vital factor in air diffusion dynamics. It is closely related to thermal turbulence and acts as a factor triggering spontaneous convection (Vinnichenko et al., 1980) and is responsible for turbulence intensity in part. In stable atmosphere conditions, mechanical turbulence becomes the main source of overall turbulence (Christen et al., 2009). Mechanical turbulence has its source in sharp-shaped obstacles, which are flown around by airstreams, as well as in moving objects, which introduce part of their kinetic energy into the air (Elterman, 1980).

The phenomena presented compare well to objects such as coking plants, characterized by high heat emission and multiplicity of spacious objects of complex shape. Some installations have significant levels of heat output and an expansive heat transfer area; for example, a coke oven battery is a low-stack emission source, usually no higher than 10 m but longer than 100 m and wider than a dozen meters or so, with a unitary heat emission approaching 3 kW/m^2^ (Żeliński et al., 2018). This is much more than the heat emission from the earth’s surface. In Central Europe, this reaches 100–130 W/m^2^ (yearly average), which is presumed in the majority of models as the factors responsible for creating turbulence in the lowest part of the earth’s atmosphere (Madany, 1996; National Research Council, 1977).

Since the coking industry implies special requirements for air quality modeling due to increased thermal and mechanical turbulence and an increase in the height of the virtual emission point over the ground, a modified model for a large Polish coking plant (called here model A) was developed taking into account all the abovementioned phenomena. As the basis for its development, the standard, first-generation Gaussian plume scheme of Pasquill type, approved for regulatory purposes (Journal of Laws, 2010), has been chosen (Model S). It contains a number of simplifying assumptions, the most important of which are continuous emission, steady-state dispersion conditions, the discreet classification of meteorological conditions, Gaussian crosswind concentration distributions, conservation of emitted masses, and total turbulent eddy deflection from the earth’s surface. The main elements distinguishing this model from others of this type are the, specific for Polish conditions, values of dispersion parameters calculated as functions of distance from the emitter, height of the virtual emission point, the state of atmosphere stability (Pasquill class), wind speed, and the ground roughness. Although being replaced nowadays by new-generation solutions (models of second generation), the models of this type are still applied in several situations—among others, where influence of the local phenomena on the atmospheric turbulence is comparable with that from sun energy (Schulman & Scire, 1980). Due to simplicity of use, existing sets of already prepared input data for majority of industrial objects, and low modeling expenses, they are also recommended for use in air management systems or for regulatory calculations. Next, the validation of this model has been performed.

The goal of the whole study was to (1) adjust the standard model to adapt it to the local, unusual diffusion conditions, significantly improving its performance (see the text above) and (2) show how validation was done with the use of SF_6_ field experiment and model quality indicators and how they behave while used for comparison of the initial and extended models.

## Materials and methods

For the purpose of increasing new model ability, the far-reaching adaptations increasing modeling accuracy for sources of high technological heat and substantial size have been applied. These changes covering modifications and corrections are presented in Żeliński et al. (2018).

As part of the model modification process, a few modules have been introduced. They take into account the following (Schulman & Scire, 1980):

- a larger rise in emitted plumes over the ground associated with considerable heat introduced into the air from technological sources (Żeliński et al., 2017),
- overlapping of plumes originating from several, closely located, neighboring sources which increases as well the height of virtual point of common emission,
- the mechanism of plume shear, which plays a particularly important role at low-altitude emission points, due to the strongly non-linear wind profile in such areas. It can influence the concentration due to locally weak air speed just over the ground surface,
- the phenomena occurring in the aerodynamic wake of buildings and other constructions, where the downwash usually causes the increase of concentration in close neighborhood of object body.

Model correction was based on the introduction of information to the model defining the increased air turbulence in the form of the adjusted values of meteorological exponent *p*. The *p* value creates the parameter reflecting specific atmosphere stability class (mainly conforming to Pasquill stability categories), i.e., describing the atmosphere thermal turbulence (Schulman & Scire, 1980; Turner, 1994). In many models, this exponent also contains a component of mechanical turbulence, which is usually taken into account by using different sets of exponential values for different types of land—typically, urban and rural (Turner, 1994). The right values of the exponent for the investigated coking plant were assessed on the basis of the vertical profiles of wind speed. For this purpose, several thousands of profiles have been taken with the use of meteorological mast situated nearby technological objects, equipped with two anemometers located at different heights. All the profiles laying in an aerodynamic wake of the plant have been analyzed after being broken down into stability classes. The values of *p* conforming in a best way the shape of profile for each class have been elaborated (Żeliński et al., 2018). Ultimate values of *p* evaluated in such manner are shown in Table 1 along with the Polish standards (Journal of Laws, 2010), and the most popular for the calculations in urban areas “urban” exponents (Schulman & Scire, 1980; US EPA, 1995).

**Table 1:** The values of exponent *p* for successive atmospheric stability classes

Type of exponent	Atmosphere stability					
	1	2	3	4	5	6
Evaluated	0.157	0.170	0.184	0.200	0.217	0.236
Polish standards	0.08	0.143	0.196	0.27	0.363	0.44
Urban	0.15	0.15	0.20	0.25	0.30	0.30

The corrected and modified model became the basis on which to develop the COPDIMO software for dispersion calculations in the coke industry.

After the changes had been done, the final validation of the elaborated model followed to confirm reaching this goal. It was expected to provide in this way quantitative data on the improvement of modeling quality compared with the standard model.

### Model validation

Statistical evaluation is being performed using a set of statistical indicators, enabling the determination of all possible shortcomings in the model. The most commonly used indicators include those developed by the US EPA (Hanna et al., 1993) and collected in the BOOT statistical model evaluation software package (ASTM, 2000; Cox & Tikvart, 1990). These indicators include the following:

➢ FB**-**fractional bias

1 $$FB=\frac{(\overline{C_o}-\overline{C_p})}{0.5\cdot(\overline{C_o}-\overline{C_p})}$$

➢ MG**-**geometric mean bias

2 $$MG=\exp(\overline{\mathrm{In}\;C_o}-\overline{\mathrm{In}\;C_p})$$

➢ NMSE**-**normalized mean square error

3 $$NMSE=\frac{\overline{{(C_o-C_p)}^2}}{\overline{C_o}\cdot\overline{C_p}}$$

➢ VG**-**geometric variance

4 $$VG=\exp\lbrack\overline{{(\mathrm{In}\;C_o-\mathrm{In}\;{\mathrm C}_{\mathrm p})}^2}\rbrack$$

*➢ R-*correlation coefficient

5 $$R=\frac{\overline{(C_o-\overline{C_o)}\cdot(C_p-\overline{C_p})}}{\sigma_{S_p}\cdot\sigma_{S_o}}\\$$

➢ FAC2**-**fraction within a factor of 2

6 $$FAC2=\frac{\mathrm{the}\_\mathrm{number}\_\mathrm{of}\;C_p:\left\{0.5\leq\frac{C_p}{C_o}\leq2\right\}}n$$

➢ FB_FN_**-**false negative

7 $$FB_{FP}=\frac{0.5\bullet\left[\left|C_{oi}-C_{pi}\right|+(C_{oi}-C_{pi})\right]}{0.5\bullet\sum_1(C_{oi}+C_{pi})}$$

➢ FB_FP_**-**false positive

8 $$FB_{FN}=\frac{0.5\bullet\sum_1\left[\left|C_{oi}-C_{pi}\right|+(C_{pi}-C_{oi})\right]}{0.5\bullet\sum_1(C_{oi}-C_{pi})}$$

The difference between FBFN and FBFP also yields the value of FB,

➢ the two**-**dimensional measure of the efficiency of the MOE (measure of effectiveness), in which the component MOE_FN_ (under prediction) measures the degree of underestimation, and MOE_FP_ (over prediction) measures the degree of the overestimation of the model in relation to the observation, based on indicators FB, FB_FN_, and FB_FP_,

9 $$MOE_{FN}=\frac{2-{\mathrm{FB}}_{\mathrm{FN}}-{\mathrm{FB}}_{\mathrm{FP}}}{2+\mathrm{FB}}$$

10 $$MOE_{FP}=\frac{2-{\mathrm{FB}}_{\mathrm{FN}}-{\mathrm{FB}}_{\mathrm{FP}}}{2-\mathrm{FB}}$$

➢ Normalized absolute difference (NAD) has also been evaluated and taken into account:

11 $$NAD=\frac{\vert\overline{C_o-C_p\vert}}{\overline{C_o}-\overline{C_p}}$$

where

*C*_*p*_-concentration evaluated by the model

*C*_*o*_-observed concentration

*n-*the size of the measurements set

*σ*_*Sp*_-the standard deviation of concentrations evaluated by the model

*σ*_*So*_-the standard deviation of the observed concentrations

In addition, three other statistical indicators adapted for validate dispersion models, i.e., the coefficient of efficiency (COE) (Legates & McCabe, 2013) and the index of accordance (IOA) (Willmott et al., 2012), have been used:

12 $$COE=1\frac{\sum i\vert C_{pi}-C_{oi}\vert}{\sum i\vert C_{oi}-\overset-C\vert}$$

13 $$\mathrm{IOA}=\left[1-\begin{array}{cc}\frac{\sum i\vert C_{pi}-C_{oi}\vert}{\sum i\vert C_{oi}-\overset-{C_o\vert}},&if\sum i\vert C_{pi}-C_{oi}\vert\leq\sum i\vert C_{oi}-\overline{C_o}\vert\\\frac{\sum i\vert C_{oi}-\overset-{C_o}\vert}{\sum i\vert C_{pi}-C_{oi}\vert}-1&if\sum i\vert C_{pi}-C_{oi}\vert>\sum i\vert C_{oi}-\overline{C_o}\vert\end{array}\right]$$

The values of the IOA index ranges from −1 to 1, while the COE index has no lower limit. A value of 1 for both IOA and COE refers to the ideal model. For air quality models assessment, IOA and COE limit values defining “good quality” models have not been set.

## Experimental

**Fig. 1:**
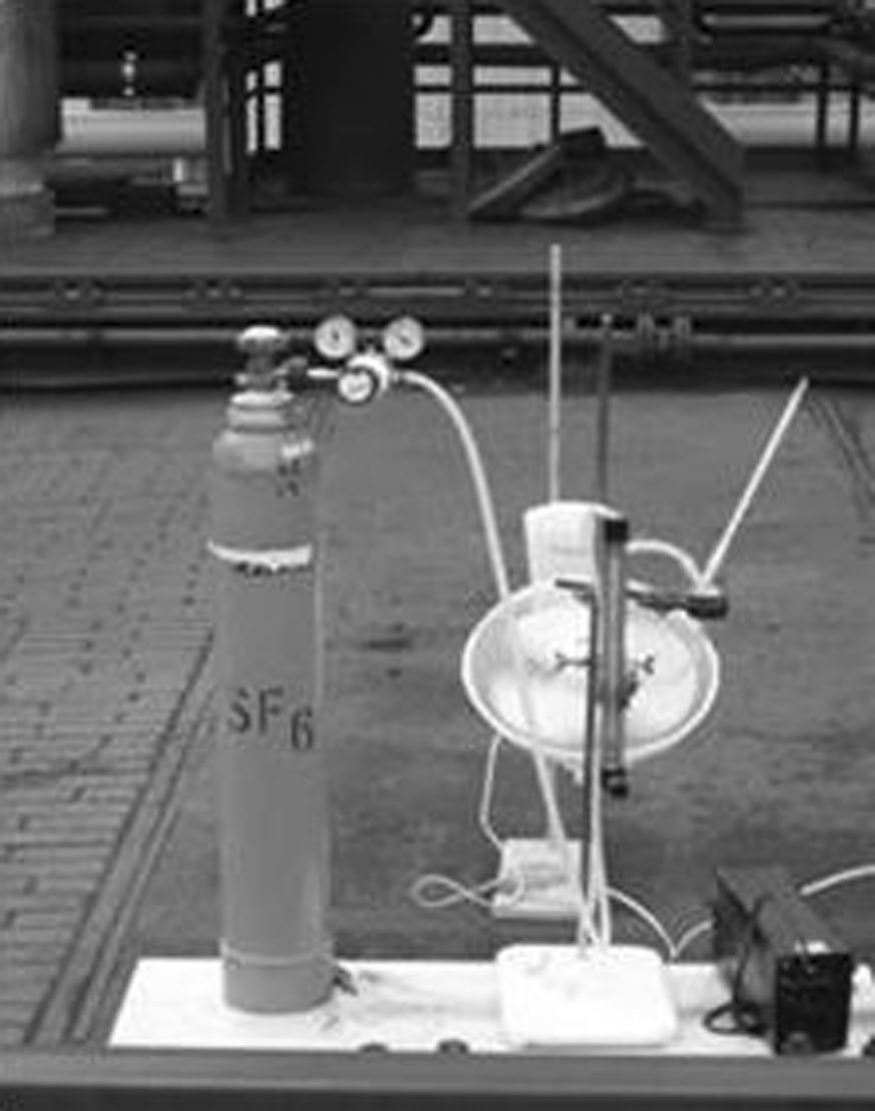
The layout used for introducing SF_6_ into the air

In order to carry out the final validation, the actual concentrations of a marker gas (SF_6_) emitted into the air from the test object were confronted, using statistical indicators, with the relevant values obtained from the model. The investigation was carried out over the entire year. A total of 24 series of marker gas concentration measurements under different weather conditions were done. To obtain concentrations in the neighborhood of the coke plant, marker gas was introduced into the air from a centrally oriented point of the coke battery for a period of 40 min. To do this in a controlled manner, the layout as shown in Fig. 1 was used, which consisted of

- a cylinder containing the SF_6_ under a pressure of about 20 atm,
- a single reducer, reducing pressure to the value of 1 – 4 atm,
- a gas output regulating valve with a rotameter calibrated on the SF_6_ (class 2.5), allowing the accurate adjustment of the marker gas flow rate,
- a mercury thermometer,
- a table ventilator ensuring the initial dilution of the marker gas in the air.

**Fig. 2:**
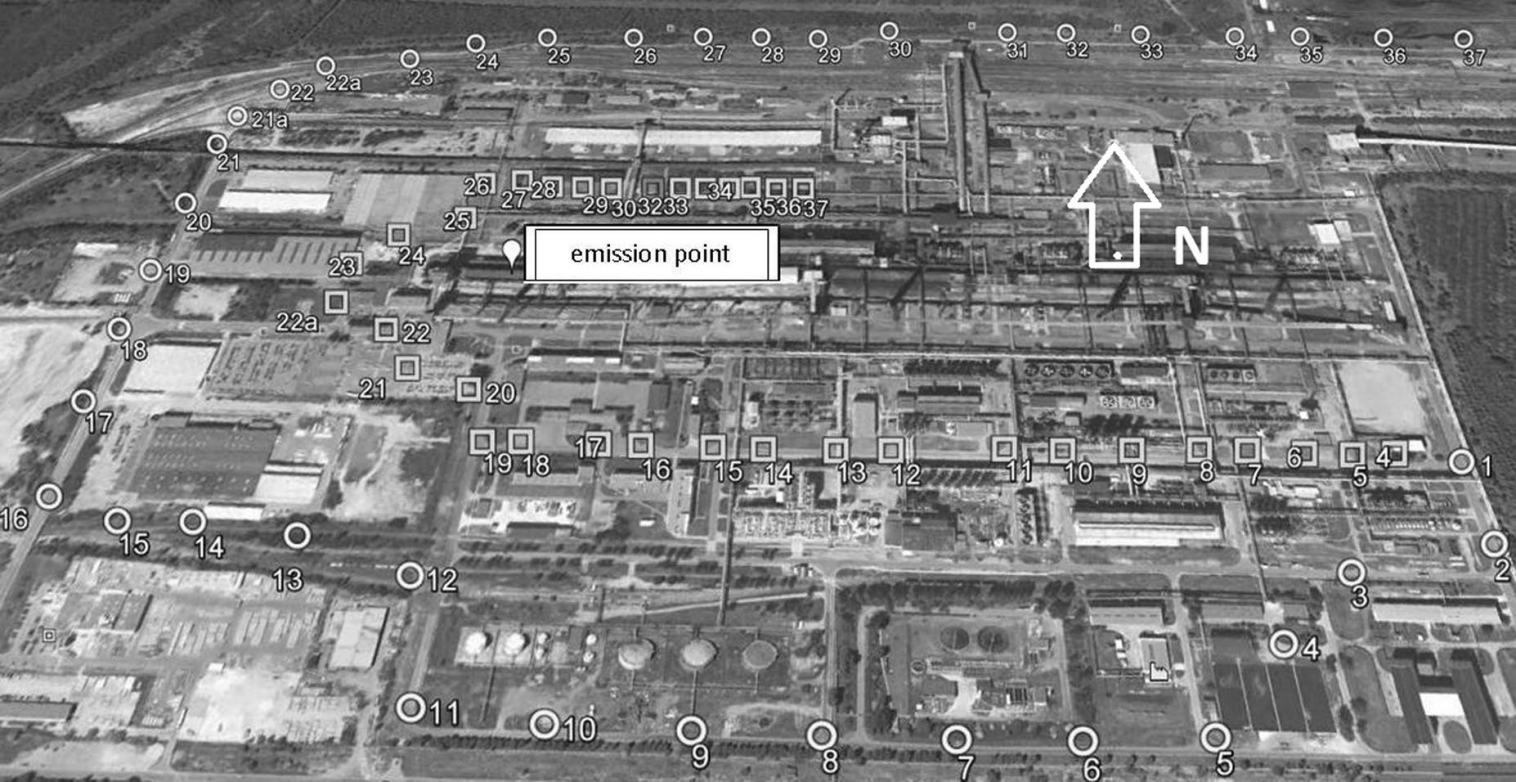
The location of measuring points on the territory of the coking plant

During each measurement, SF_6_ concentrations in the air were measured at 14 points on two measurements axes (seven on each): internal, located closer to the emission point (squares in Fig. 2), and outer, more remote from the point of emission (circles in Fig. 2).

These points were chosen out of 74 receptors prepared in advance in the close neighborhood of the factory, along two axes lying at a distance of respectively 200–400 m and 800–1200 m from the source of emission. Such distance ensured that the gap from each receptor to the emission source was greater than the theoretical occurrence distance of the maximum concentration for this source. All the 14 measurement points for each experiment were chosen with respect to the actual wind direction, in order to embrace the whole width of the SF_6_ plume by measuring points. Due to localization problems, there was a lack of receptors on the eastern side of the coking plant (Fig. 2).

The measurement equipment was situated each time in pre-prepared locations so that they were on the leeward side of the emission point. This assumption was checked with the meteorological observations. The investigation was carried out over the period October 2012–September 2013. It consisted of 24 series of measurements in conditions covering wind speed up to 3.1 m/s and atmosphere stability classes A, B, and D (US EPA, 2000). For the formal reasons, the presence of measurement equipment on a territory of the coking plant was able from the late morning to early afternoon, which made classes F and F unobtainable. As for the stability C, only one measurement has been done in this class—it was excluded from further proceeding.

Air containing SF_6_ was taken to the sampling bags made of chemically resistant FlexFoil material with a capacity of 10 dm^3^. Samples were taken with an adjustable, automatically maintained flow. Each bag, along with the aspirator, was placed on a tripod at a height of about 1.8 m. The sampling time was established as 20 min. Samples were analyzed in order to determine the concentrations of SF_6_. The determination was performed using a gas chromatograph with an electron capture detector (ECD). Under ideal conditions, the ECD allows for SF_6_ concentration measurements in gas mixtures at a level up to 1 ppt (Śliwka, 2003).

## Results and discussion

**Fig. 3:**
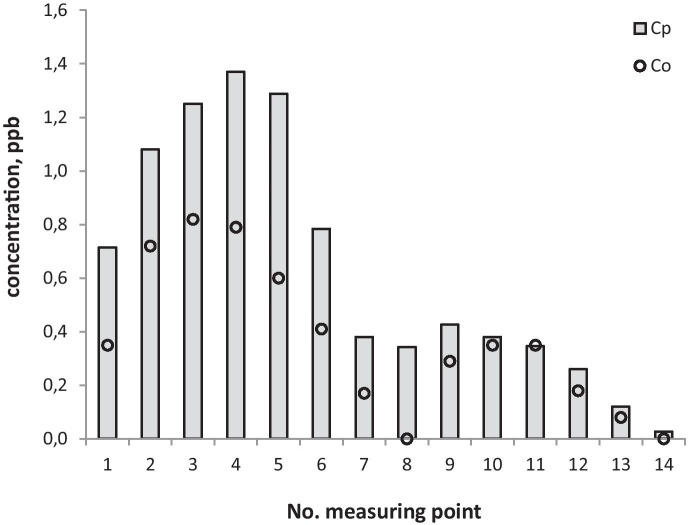
SF_6_ concentrations observed (*C*_*o*_) and calculated with COPDIMO (*C*_*p*_) during experimental conditions: stability class = D, wind speed = 2.1 m/s

**Fig. 4:**
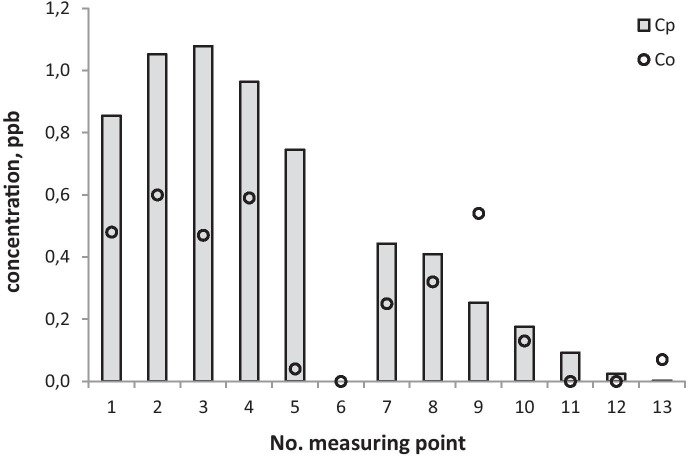
SF_6_ concentrations observed (*C*_*o*_) and calculated with COPDIMO (*C*_*p*_) during experimental conditions: stability class = D, wind speed = 2.5 m/s

**Fig. 5:**
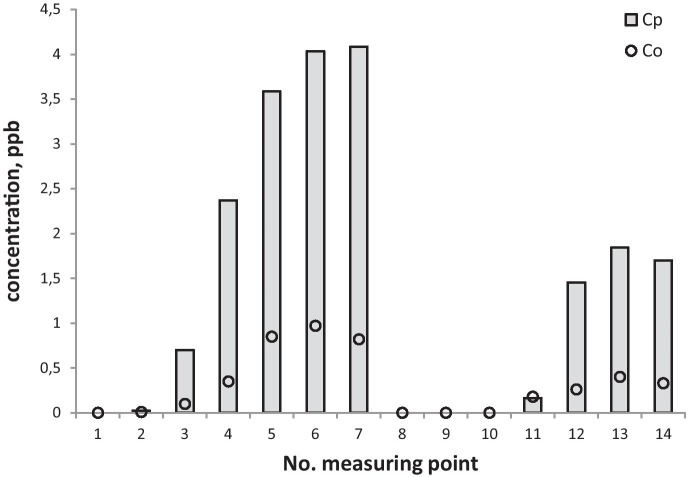
SF_6_ concentrations observed (*C*_*o*_) and calculated with COPDIMO (*C*_*p*_) during experimental conditions: stability class = B, wind speed = 1.1 m/s

In order to validate the conformity and quality of the COPDIMO program, a validation process was performed. The examples of observed (*C*_*o*_) and calculated (*C*_*p*_) SF_6_ concentration distributions on the measuring axes—internal (points 1–7) and outer (points 8–14)—are presented in Figs. 3, 4, and 5.

Table 2 presents the statistical indicators calculated for the developed model (model A) and for the standard model (model S), commonly utilized in Poland (Journal of Laws, 2010).

**Table 2:** The values of particular statistical indicators calculated for both models: S and A

Indicator	Concentration observed	Model S	Model A
FB	-	−1.57	−1.02
MG	-	0.19	0.46
NMSE	-	35.08	7.35
VG	-	186.36	36.04
R	-	0.539	0.674
FAC2	-	0.081	0.224
NAD	-	0.811	0.656
Mean	0,42	4.44	1.54
*σ*—standard deviation	0,76	9.88	2.56
maximum	4,31	78.75	19.17
FB_FN_	-	0.025	0.144
FB_FP_	-	1.598	1.168
MOE_FN_	-	0.974	0.847
MOE_FP_	-	0.093	0.232
IOA		−7.54	−2.04
COE		−0.88	−0.67

In addition, the measurement data obtained during the field experiment were grouped by stability classes and wind speed ranges. In order to compare results of modeled values with measured ones, selected values of model evaluation parameters were determined, i.e., FB, NMSE, FAC2, NAD, MG, IOA, and COE for particular combinations of wind speed and atmosphere stability classes. The analysis was limited to 3 most frequent wind speed ranges as variants *u1*, *u2*, *u3*:

- *u* ≤ 1 m/s (*u1*),
- 1 < *u* ≤ 2 m/s (*u2*),
- 2 < *u* ≤ 3 m/s (*u3*),

and 3 classes of atmosphere stability according to the Pasquill scale: A, B, and D.

**Fig. 6:**
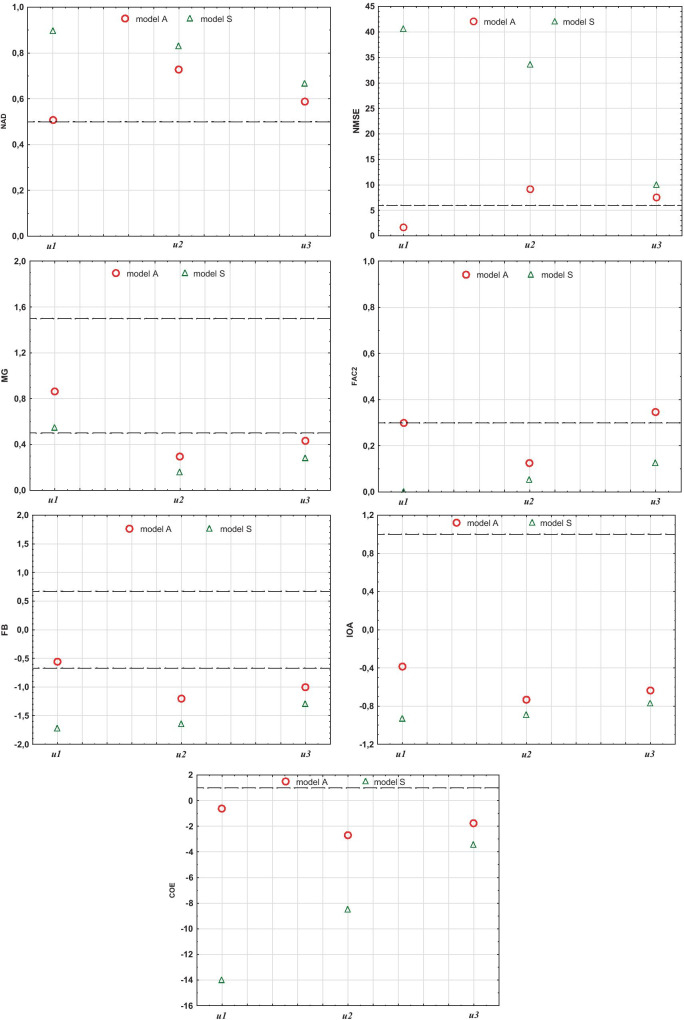
Graphical presentation of models S and A statistical indicators: NAD, NMSE, MG, FAC2, FB, IOA, and COE, obtained for various wind speeds

**Fig. 7:**
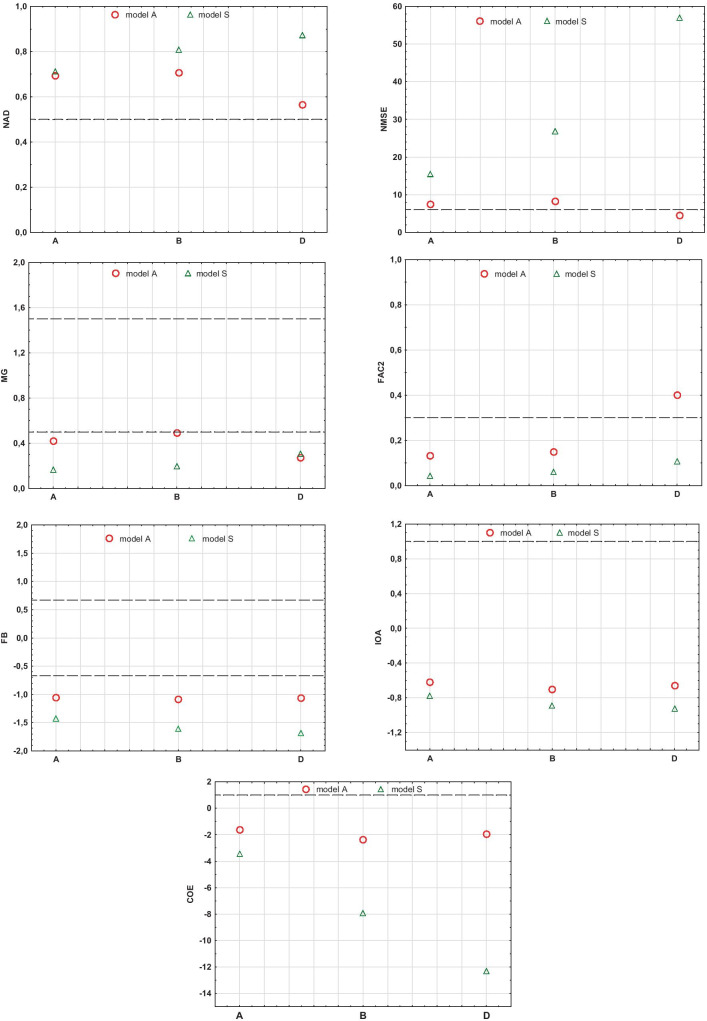
Graphical presentation of models S and A statistical indicators: NAD, NMSE, MG, FAC2, FB, IOA, and COE, obtained for various atmosphere stability classes

The results of such analysis are presented in Figs. 6 and 7.

**Fig. 8:**
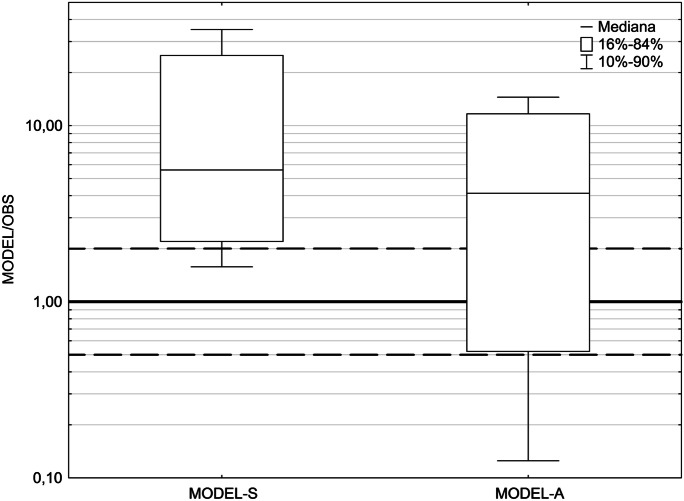
A box and whisker chart of concentrations calculated with models S and A, related to the concentrations measured (the upper dotted line refers to double overestimation and the lower dotted line refers to double underestimation)

**Fig. 9:**
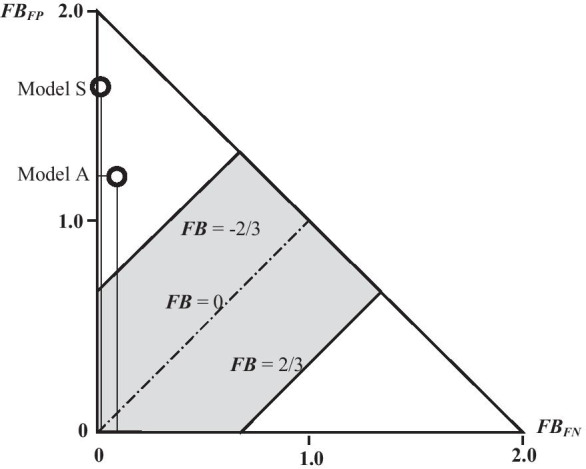
A diagram showing the relationships between FB, FB_FN_, and FB_FP_ with an indication of the values of the fractional bias FB obtained for models S and A (black circles)

To analyze the quality of models A and S, a box and whisker charts were developed as well as a diagram showing the relationships between FB, FB_FN_, and FB_FP_ (Fig. 8 and 9).

The part of the chart in gray (Fig. 9) is the area between the double overestimation and the double underestimation, relative to the concentrations observed (Chang, 2003; Chang & Hanna, 2004). The hypotenuse line is a collection of points that describe a situation in which complete disjoint concentrations are observed, with a prognosis obtained from the program, regardless of other statistical parameters.

On the basis of field experiments, one can notice that model A differs in an essential way from the standard one (Figs. 3, 4, and 5). It is especially important for stable atmosphere, which usually creates the most critical conditions for the final environmental impact of the coke industry (due to the presence of strong emission flows of small height). In this case, the comparison of concentrations (Figs. 3, 4, and 5) and indicators (Table 2; Figs. 6 and 7) show that concentrations obtained by calculations made with the developed model, clearly exhibit lower and more realistic values. This allows us to declare that model A creates a better tool for assessing the influence of a coking plant on air contamination compared with the standard one.

On the other hand, taking into account the values of the indicators reflecting the perfect fitted model (Table 3), the indicators for FB, FB_FN_, and FB_FP_ obtained for models S and A reveal a tendency among both models to overestimate the concentration evaluations. Such behavior, subject to minor exceedances, is however seen as desirable when it comes to models of this type.

**Table 3:** A compilation of statistical indicators values, corresponding to the perfect model (Cox & Tikvart, 1990)

Indicator	Underestimation	Value for perfect model	Overestimation
FB	< 0	0	> 0
MG		1	
NMSE		0	
VG		1	
R		1	
FAC2		1	
FB_FN_		0	
FB_FP_		0	
MOE_FN_		1	
MOE_FP_		1	
*σ*—standard deviation		0	

The exceedance of the double overestimation value, which creates the upper limit of the model considered to be perfect, reaches about 75% of this value in the case of model A and about 150% in the case of Model S (Fig. 8). The indicators MOE_FN_ and MOE_FP_, which are closely related to FB, FB_FN_, and FB_FP_, clearly confirm a smaller tendency on the part of model A to overestimate the concentrations in comparison to model S. The MG indicator, which reflects the level of derogations from the average of the values observed, is respectively 0.19 and 0.46 for models S and A (Table 2). This means that the MG for model A is much closer to unity (desired value) than in the case of model S. A similar trend shows a VG indicator, whose values are 186.36 for model S and 36.04 for model A.

The FAC2 indicator, which describes the percentage of calculated concentrations contained in the range of 0.5 to 2, in relation to the concentration observed, is 8% for model S and about 22% for Model A. The correlation coefficient *R* takes a generally lower value for model S (0.539) than for model A (0.674) (Table 2).

Between the indicators shown, the NMSE creates the basic, cumulated parameter which describes the tendencies of the model to both overestimate and underestimate concentrations, as well as the overall quality of the modeling. For this reason, it is widely used by many authors as a universal quality indicator of modeling (Chang, 2003; Irwin et al., 2002, 2003). Its value for models S and A, as well as for obtained for a few other popular dispersion models in the field experiments, is shown in Table 4.

**Table 4:** Comparison of the *NMSE* indicator for different dispersion models (Irwin et al., 2002)

Model	Experiment			
	Prairie Grass	EPRI Kincaid	EPRI Indianapolis	Coking plant
AERMOD	1.144	0.400	0.368	-
HPDM	6.676	0.298	0.361	-
ISC3	4.581	1.652	0.479	-
Model A	-	-	-	7.35
Model S	-	-	-	35.08

In the new generation models of general purpose, as pointed here AERMOD or ADMS, the discrete classification of meteorological conditions used in traditional models has been abandoned in favor of a continuous dependence of plume parameters (dispersion, elevation, asymmetry) on meteorological parameters (thickness of the mixing layer, vertical profile of wind speed including twist with height, characteristics of atmospheric states including their vertical variation). The similarity theory and boundary layer parameters (surface heat flux, surface momentum flux, boundary layer thickness of the atmosphere) are used here to describe the structure of the atmospheric boundary layer. These values are obtained usually with the help of meteorological preprocessors, which however requires hourly data from a meteorological station and twice-daily determined vertical profile data of wind speed and temperature from a radiosonde station or meteorological mast. Such improvements allow to significantly increase modeling accuracy as well as the range of its applicability compared with the models of the first generation, despite retaining the Gaussian character of the concentration distribution (at least for neutral and stable conditions). These models have the usual ability to incorporate directly or indirectly the amount of sensible heat into calculations, which conveniently allows for the introduction of the effect of technological heat on the dispersion. The extended models of the new generation are enriched with modules designed to handle specific propagation conditions. For instance the Hybrid Plume Dispersion Model (HPDM), which was developed for handling tall stacks, has deepened meteorological component based on observational and modeling studies of the planetary boundary layer. For this reason, it is found to be an improvement over the standard models especially for light-wind convective conditions and high-wind neutral conditions. In turn, mentioned here is the ISC model (Industrial Source Complex Model) which is an old-fashioned, steady-state Gaussian plume model of first generation designed for assessing concentrations of non-reactive pollutant emitted from a wide variety of industrial complex sources. It has numerous options for computing such important phenomena as a dry deposition of the pollutant, the hydrodynamic effects on plume effective height, or the cumulative impact of various types of sources, and is specifically aimed at taking into calculation the effect of stacks height on the behavior of the pollutant plume. Taking this information under consideration, one can expect far better behavior from mentioned models, especially of the new generation, over the traditional ones, like model A or S. These predictions are partially reflected in the comparison presented (Table 4). On the other hand, a relatively good performance of ISC3 compared with new-generation models and not far worse of model A shows that the old generation but vastly improved model can produce quite reasonable results, slightly only different to those delivered by new-generation ones.

In the case of models S and A, the value of the NMSE indicator is higher than for the compared models and highly different: respectively, 35.08 and 7.35 (Table 4). This indicates that model A is clearly although not extremely worse than other models enumerated in Table 4 but at the same time far better fitted to actual dispersion conditions than model S, which has been used so far.

On the box and whisker charts (Fig. 8), statistical parameters relating to the quotients of calculated and observed concentrations are presented: the median (Me), the percentiles 16% and 84% determining the value range Me ± *σ* (where *σ* stands for standard deviation) and the standard percentiles 10% and 90%. The analysis of charts makes it possible to declare that the chart describing model A has clearly shifted in the direction of the symmetry axis (the bold line with a value of 1.00), thus illustrating a better fit compared with model S.

The medians, based on the entire set of concentrations, have shifted relative to each other: their value is about 4 for model A and about 5.6 for model S (Fig. 8). In addition, the box frame for model A partly lies above the area corresponding to the double overestimation or underestimation (doted lines with values of 0.50 and 2.00) and partly overlaps it. The position of the lower edge of the box frame reaching the bottom dotted line means that about 16% of the calculated concentrations are more than double underestimated. Meanwhile, for model S, both of these areas are disjoint areas, while the degree of double underestimation is less than 10%. At the same time, the degree of overestimation for this model is clearly higher: over 84% of the calculated values are overestimated by more than 200%.

The values of three other indicators, MG, VG, and FAC2, have also been calculated for three popular models: ISC3, ADMS, and AERMOD (Hanna et al., 2001) on the base of 5 field experiments covering conditions typical of the applications of these models. The results are shown in Table 5 along with the same indicators evaluated for model S and model A on the base of the presented experiment.

**Table 5:** Indicators obtained for 3 models: ISC3, ADMS, and AERMOD in field experiments and for model S and model A in the presented work

	ISC3	ADMS	AERMOD	Model S	Model A
MG	0.70	1.22	1.70	0.19	0.46
VG	7.70	2.40	2.90	186.3	36.04
FAC2	0.33	0.53	0.46	0.081	0.224

The values in Table 5 show the clearly higher quality of model A over S and visible worse of the least one in compare with ADMS and AERMOD as well as ISC3. Judging the values of evaluated statistical indicators, one should take into consideration different conditions in which the data for such evaluations were acquired: on the one side, the field experiment with all diffusive parameters relatively stable in time and space; on the other side, the complex industrial environment undergoing to fast and rather radical changes. Such a meaningful difference in the experimental conditions leads to a belief that the actual difference between model A and the models may be less than it is indicated by cited numbers.

The basic criteria that should be met by the air quality dispersion model were also specified in the works of Chang and Hanna (2004) and Hanna and Chang (2012), with strict distinction between rural and urban areas. The acceptation criteria for a model designated for the urban area can be defined as follows:

- |FB|< 0.67, which means that the relative average deviation should be less than 2.0
- NMSE < 6.0, that is, random dispersion of results is approx. 2.4 times the average value
- FAC2 > 0.3, which means that at least half of the results are within the range 0.3 ≤ *C*_p_/*C*_o _≤ 3.0
- The average error is in the range ± 50%, that is, NAD < 0.5 and 0.5 < MG < 1.5

When the model is used for urban environment, its expected performance is not as good as in rural areas due to the disturbances introduced by buildings and other forms of land development. This is reflected by the less restricted model acceptance criteria—about 2 times compared with the criteria for rural cases. And so the acceptance criteria for the rural area model are as follows:

- |FB|< 0.3, which means that the relative average deviation should be less than 0.3
- NMSE < 3.0, that is, random dispersion of results is approx. 1.7 times the average value
- FAC2 > 0.5, which means that at least half of the results are within the range 0.5 ≤ *C*_p_/*C*_o_ ≤ 2.0
- The average error is in the range ± 30%, that is, NAD < 0.3 i 0.7 < MG < 1.3

Given the strong air turbulence caused by the additional heat introduced into the air and the impact of objects of significant dimensions forming an industrial infrastructure, urban criteria should be considered as appropriate benchmarks.

The values of indicators FB, NMSE, FAC2, NAD, and MG creating the acceptation criteria and calculated for model S and model A are presented in Table 6.

**Table 6:** Accepted ranges and values calculated for model S and model A of indicators: FB, NMSE, FAC2, NAD, MG

	Rural	Urban	Model S	Model A
|FB|	< 0.3	< 0.67	1.57	1.02
NMSE	< 3.0	< 6.0	35.08	7.35
FAC2	> 0.5	> 0.3	0.081	0.224
NAD	< 0.3	< 0.5	0.811	0.656
MG	0.7 < MG < 1.3	0.5 < MG < 1.5	0.19	0.46

**Fig. 10:**
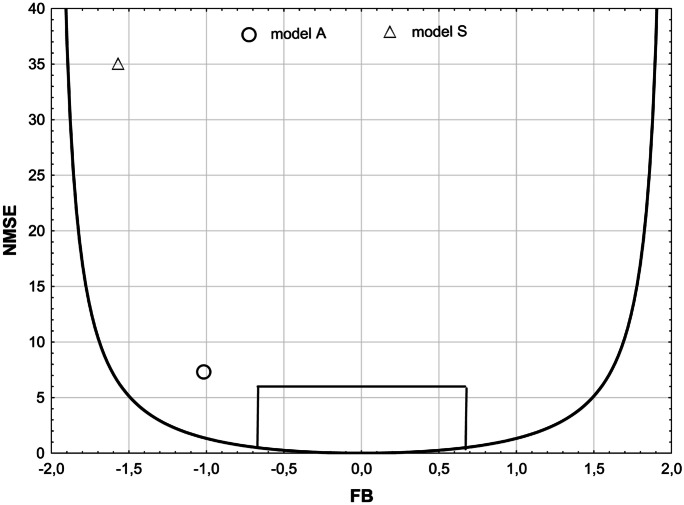
FB dependencies on NMSE calculated for models A and S. Parabola indicates the minimum NMSE for a given FB

In Fig. 10, the dependence between the systematic error FB and the normalized mean square error NMSE for both models is presented in a graphical form. The line reflecting the minimum value of the normalized mean square error NMSE_min_, has been determined according to the formula:

14 $${\mathrm{NMSE}}_{\mathrm{min}}=\frac{4{\mathrm{FB}}^{2}}{4-{\mathrm{FB}}^{2}}$$

In Fig. 10, the area bounded with a parabola from the bottom and with a fragment of a rectangle from above predetermines the best-fit area for urban criteria.

For the ideal model FB = NMSE = 0. The data presented in Fig. 10 suggests that model A has a lower relative mean deviation (FB) and smaller dispersion (NMSE).

The content of Table 6 and Fig. 10 once more shows the higher quality of model A over S, revealed by values of indicators closer to the desirable ranges for model A. In this case, all indicators also lie outside these ranges, showing how industrial conditions are difficult to be modeled compared with urban or rural cases.

All presented indicators, diagrams, and charts ultimately describe the same model feature—the modeling accuracy—but the conclusions that can be drawn from them are not equally clear. The assessment of the indicators usefulness can be carried out on the basis of the presented experiment by analyzing their calculated values for models S and A. The introduction of additional turbulence to the flow, associated with technology, results in different amounts of modeling errors depending on wind speed and atmosphere stability. The higher the wind speed, the greater the mechanical turbulence and the proportionally smaller the share of thermal turbulence in the total value. Similarly, under convective conditions, the share of thermal turbulence is less than at neutral stability. This means that the improved model should show its superiority over the traditional one particularly in the field of low wind speeds and neutral stability, and such trends should be indicated by individual indicators. The analysis of their values for both models shows that with respect to wind speed, the described tendency can be most noticeable for the range *u1* of wind speed (Fig. 6). The whole range *u1–u3* is particularly best described by NMSE and COE while NAD, FB, and IOA also describe it well although in a less convincing way, especially due to minor differences for the range *u2*. On the other hand, it is difficult to treat MG and FAC2 values as a good reflection of such relationship. When assessing atmosphere stability, the described relationship is best demonstrated by NMSE and COE (Fig. 7). The indications of the other indicators are not so consistent although generally correct, with the exception of *NAD* and MG which underestimate the differences between models in stability A, showing the same value. Ultimately, NMSE and COE appear to be the best indicator of the model quality.

When it comes to the deeper analysis, the two-dimensional measures FB_FN_, FB_FP_, MOE_FN_, and MOE_FP_ can be used, as well as a diagram showing the relationships between FB, FB_FN_, and FB_FP_ (Fig. 9), traditional box and whisker chart of concentrations calculated with both models (Fig. 8), and also a chart showing FB dependencies on NMSE (Fig. 10).

The best tool for a more comprehensive assessment of the quality of the model seems to be the box and whisker chart and also the chart showing FB dependencies on NMSE. The box and whisker chart enable an extended analysis of the distribution of values generated by a model against reference quantities and show the percentage content of obtained concentrations in the scope of double overestimation and underestimation. The graph of NMSE dependence on FB simultaneously allows the analysis of two important statistical indicators and checking if they fall within the specified limits.

## Conclusions

The main idea of this paper was to show the whole model validation workflow with the use of indicators for confirmation that the adaptation of a standard model to accommodate local, unusual diffusion conditions has been successful. It was done for the case of the coking industry, where impacts of phenomena characteristic for such objects are very strong, changing the dispersion conditions in a significant way. To achieve this goal, they were recognized and analyzed. On such basis of the standard, a universal model has been extended incorporating modules describing the main of them. Several quality indicators were calculated for the initial and final models, allowing comparison of their output with the results of a field experiment with controlled SF_6_ dispersion. All of the indicators calculated have shown supremacy of the second one.

In this way also, the phenomena assumed as characteristic for coke industry and introduced into modeling—a larger rise in plumes over the ground, overlapping of plumes from neighboring sources, the dominating mechanism of plume shear at low-altitude emission points, strong building downwash occurring in the aerodynamic wake of big-sized industrial constructions, and the increased atmosphere turbulence of thermal and mechanical origin—have been confirmed as having a high effect on pollutant dispersion in the air. Thus, one can say that dispersion modeling made for each industrial plant, in particular characterized with high technological heat emission, should reflect the impact of these phenomena on calculation results. This can be achieved by introducing into the model suitable adjustments relating to the individual conditions, which demands preparing corrections for every particular object or, at least, the group of objects. This is labor-intensive but worth the use—it significantly raises the quality of the modeling results, which is shown by very different values of comparable indicators in the presented case. This rise may not be too spectacular, especially if it is compared with the results obtained in field experiments under thoroughly controlled, prepared conditions, but it touches mainly high concentrations on the basis of which environmental decisions are made.

Not only should the statistical indicators of the model performance be used after the model had been modified in order to confirm its final suitability for the calculations on the field of air quality assessment, but also they can serve as a basis for selecting the best modeling variant during the model preparation, if any alternative is possible for a particular case.

From the point of view of the working properties, NMSE and COE seem to be the best indicators of a simple type, which show the model’s quality in a straight, convinced way. In a group of the higher-complexity tools which are the box and whisker chart, they enable the extended analysis and more elaborated conclusions as the result of models investigation can be pointed.

Based on the experience gained, some recommendations can be made for future validation works. It would be highly advisable to automate the marker gas emission and concentration measurements in order to increase the number of measurements and allow them to be performed throughout the night (the presence of persons beyond the basic staff in the night is usually forbidden on many installations). This will allow for capturing the effects on concentrations of weakened temperature inversions above the industrial plant. Also, the use of a second measurement point (or more) would allow for a significant increase in the number of measurements made with unchanged tracer emissions (the emission of SF_6_, as a greenhouse gas, is subject to strong limitations).

When validating the model, the quality of meteorological data is extremely important and has a great influence on the calculation results, which in the validation process are then confronted with measured values. Therefore, meteorological measurements should take into account an extended range of parameters relevant for modeling, which in particular concerns solar radiation and the height of the mixing layer above and around the object under study.
